# 2-(Benzyl­sulfan­yl)pyridine *N*-oxide

**DOI:** 10.1107/S1600536808011446

**Published:** 2008-04-26

**Authors:** B. Ravindran Durai Nayagam, Samuel Robinson Jebas, H. Johnson Jeyakumar, Dieter Schollmeyer

**Affiliations:** a Department of Chemistry, Popes College, Sawyerpuram 628 251, Tamil Nadu, India; b Department of Physics, Karunya University, Karunya Nagar, Coimbatore 641 114, India; c Department of Physics, Popes College, Sawyerpuram 628 251, Tamil Nadu, India; d Institut für Organische Chemie, Universität Mainz, Duesbergweg 10-14, 55099 Mainz, Germany

## Abstract

In the title compound, C_12_H_11_NOS, the dihedral angle between the oxopyridinium and phenyl rings is 58.40 (1)°. The crystal structure is stabilized by C—H⋯O hydrogen bonds, π–π stacking inter­actions involving the pyridinium rings [centroid–centroid distance = 3.6891 (9) Å] and C—H⋯π inter­actions.

## Related literature

For bond-length data, see: Allen *et al.*(1987 ▶). For biological activities of *N*-oxide derivatives, see: Bovin *et al.* (1992 ▶); Katsuyuki *et al.* (1991 ▶); Leonard *et al.* (1955 ▶); Lobana & Bhatia (1989 ▶); Symons & West (1985 ▶). For related literature, see: Jebas *et al.* (2005 ▶); Ravindran *et al.* (2008 ▶).
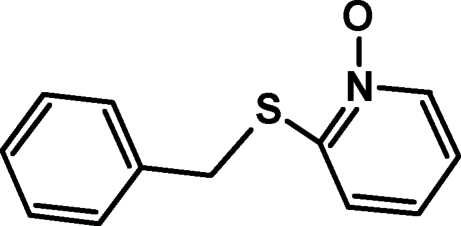


## Experimental

### 

#### Crystal data

C_12_H_11_NOS
*M* *_r_* = 217.28
Monoclinic, 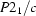

*a* = 5.7277 (2) Å
*b* = 15.8760 (3) Å
*c* = 11.6498 (4) Å
β = 97.816 (2)°
*V* = 1049.51 (6) Å^3^
*Z* = 4
Cu *K*α radiation
μ = 2.49 mm^−1^
*T* = 298 (2) K
0.6 × 0.32 × 0.16 mm

#### Data collection

Enraf–Nonius CAD-4 diffractometer
Absorption correction: numerical (*CORINC*; Draeger & Gattow, 1971 ▶) *T*_min_ = 0.423, *T*_max_ = 0.676
2183 measured reflections
1979 independent reflections
1865 reflections with *I* > 2σ(*I*)
*R*_int_ = 0.020
3 standard reflections frequency: 60 min intensity decay: 3%

#### Refinement

*R*[*F* ^2^ > 2σ(*F* ^2^)] = 0.032
*wR*(*F* ^2^) = 0.088
*S* = 1.05
1979 reflections
137 parameters
H-atom parameters constrained
Δρ_max_ = 0.23 e Å^−3^
Δρ_min_ = −0.23 e Å^−3^

### 

Data collection: *CAD-4 Software* (Enraf–Nonius, 1989 ▶); cell refinement: *CAD-4 Software*; data reduction: *CORINC* (Draeger & Gattow, 1971 ▶); program(s) used to solve structure: *SHELXS97* (Sheldrick, 2008 ▶); program(s) used to refine structure: *SHELXL97* (Sheldrick, 2008 ▶); molecular graphics: *SHELXTL* (Sheldrick, 2008 ▶) and *PLATON* (Spek, 2003 ▶); software used to prepare material for publication: *SHELXL97*.

## Supplementary Material

Crystal structure: contains datablocks global, I. DOI: 10.1107/S1600536808011446/ci2583sup1.cif

Structure factors: contains datablocks I. DOI: 10.1107/S1600536808011446/ci2583Isup2.hkl

Additional supplementary materials:  crystallographic information; 3D view; checkCIF report

## Figures and Tables

**Table 1:** Hydrogen-bond geometry (Å, °)

*D*—H⋯*A*	*D*—H	H⋯*A*	*D*⋯*A*	*D*—H⋯*A*
C5—H5⋯O7^i^	0.93	2.42	3.323 (2)	164
C14—H14⋯*Cg*1^ii^	0.93	2.92	3.560 (2)	127
C4—H4⋯*Cg*2^iii^	0.93	2.99	3.777 (2)	143

Symmetry codes: (i) 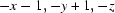; (ii) 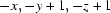; (iii) 
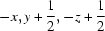
. *Cg*1 is the centroid of the ring C1–C5/N6 and *Cg*2 is the centroid of the ring C10–C15.

**Fig. 1.**
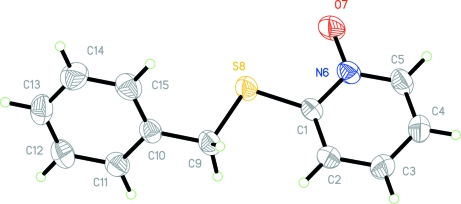
The molecular structure of the title compound, showing 50% probability displacement ellipsoids and the atomic numbering scheme.

**Fig. 2.**
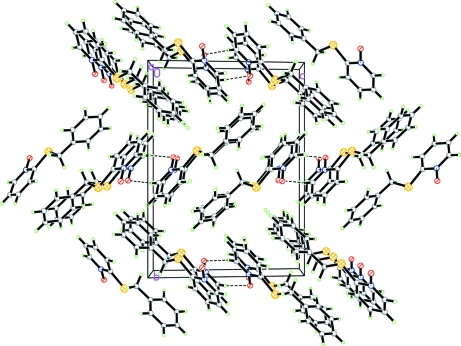
The crystal packing of the title compound, viewed along the a axis. Hydrogen bonds are shown as dashed lines.

## supplementary crystallographic information

### Comment

N-Oxides and their derivatives show a broad spectrum of biological activity, such as antifungal, antibacterial, antimicrobial and antibacterial activities (Lobana & Bhatia, 1989; Symons *et al.*, 1985). These compounds are also found to be involved in DNA strand scission under physiological conditions (Katsuyuki *et al.*, 1991; Bovin *et al.*, 1992). Pyridine N-oxides bearing a sulfur group in position 2 display significant antimicrobial activity (Leonard *et al.*, 1955). In view of the importance of N-oxides, we have previously reported the crystal structures of N-oxide derivatives (Jebas *et al.*, 2005; Ravindran Durai Nayagam *et al.*, 2008). As an extension of our work on these derivatives, we report here the crystal structure of the title compound (Fig. 1).

The bond lengths and angles agree well with the N-oxide derivatives reported earlier (Jebas *et al.*, 2005; Ravindran Durai Nayagam *et al.*, 2008). The N—O bond length is in good agreement with the mean value of 1.304 (15) Å reported in the literature for pyridine N–oxides (Allen *et al.*,1987).

The oxopyridinium and benzene rings are planar to within ±0.002 (2) Å and ±0.005 (2) Å, respectively, and they form a dihedral angle of 58.40 (1)°. Atom O7 deviates from the plane of the pyridinium ring by -0.012 (1) Å.

In the crystal structure, inversion related molecules at (x, y, z) and (-1-x, 1-y, -z) are linked by C—H···O hydrogen bonds to form a *R*_2_2(8) ring (Fig. 2). In addition, the crystal packing is stabilized by a π-π interaction between the pyridinium rings of adjacent molecules at (x, y, z) and (-x, 2-y, -z), with a ring centroid to centroid distance of 3.6891 (9) Å. Weak C—H···π interactions involving the two aromatic rings are also observed.

### Experimental

A mixture of benzyl chloride, (0.126 g, 1 mmol) and 1-hydroxypyridine-2-thione sodium salt (0.149 g, 1 mmol) in water and methanol (30 ml each) was heated at 333 K with stirring for 30 min. The compound formed was filtered off, and dried (0.20 g, 92%). The compound was recrystallized from chloroform-methanol (1:1 *v*/*v*).

### Refinement

H atoms were positioned geometrically [C-H = 0.93 (aromatic) or 0.97 Å (methylene)] and refined using a riding model, with *U*_iso_(H) = 1.2*U*_eq_(C).

### Crystal data

**Table:**

C_12_H_11_NOS	*F*_000_ = 456
*M**_r_* = 217.28	*D*_x_ = 1.375 Mg m^−^^3^
Monoclinic, *P*2_1_/*c*	Cu *K*α radiation λ = 1.54178 Å
Hall symbol: -P 2ybc	Cell parameters from 25 reflections
*a* = 5.7277 (2) Å	θ = 65–70º
*b* = 15.8760 (3) Å	µ = 2.49 mm^−^^1^
*c* = 11.6498 (4) Å	*T* = 298 (2) K
β = 97.816 (2)º	Plate, colourless
*V* = 1049.51 (6) Å^3^	0.6 × 0.32 × 0.16 mm
*Z* = 4	

### Data collection

**Table:**

Enraf–Nonius CAD-4 diffractometer	*R*_int_ = 0.020
Monochromator: graphite	θ_max_ = 69.9º
*T* = 298(2) K	θ_min_ = 4.7º
ω/2θ scans	*h* = 0→6
Absorption correction: numerical(CORINC; Draeger & Gattow, 1971)	*k* = 0→19
*T*_min_ = 0.423, *T*_max_ = 0.676	*l* = −14→14
2183 measured reflections	3 standard reflections
1979 independent reflections	every 60 min
1865 reflections with *I* > 2σ(*I*)	intensity decay: 3%

### Refinement

**Table:**

Refinement on *F*^2^	H-atom parameters constrained
Least-squares matrix: full	*w* = 1/[σ^2^(*F*_o_^2^) + (0.0468*P*)^2^ + 0.3205*P*] where *P* = (*F*_o_^2^ + 2*F*_c_^2^)/3
*R*[*F*^2^ > 2σ(*F*^2^)] = 0.032	(Δ/σ)_max_ = 0.001
*wR*(*F*^2^) = 0.088	Δρ_max_ = 0.23 e Å^−^^3^
*S* = 1.05	Δρ_min_ = −0.23 e Å^−^^3^
1979 reflections	Extinction correction: SHELXL97 (Sheldrick, 2008), Fc^*^=kFc[1+0.001xFc^2^λ^3^/sin(2θ)]^-1/4^
137 parameters	Extinction coefficient: 0.0138 (9)

### Special details

**Table:**

Geometry. All e.s.d.'s (except the e.s.d. in the dihedral angle between two l.s. planes) are estimated using the full covariance matrix. The cell e.s.d.'s are taken into account individually in the estimation of e.s.d.'s in distances, angles and torsion angles; correlations between e.s.d.'s in cell parameters are only used when they are defined by crystal symmetry. An approximate (isotropic) treatment of cell e.s.d.'s is used for estimating e.s.d.'s involving l.s. planes.

### Fractional atomic coordinates and isotropic or equivalent isotropic displacement parameters (Å^2^)

**Table:**

	*x*	*y*	*z*	*U*_iso_*/*U*_eq_	
C1	0.0305 (2)	0.50000 (9)	0.21475 (12)	0.0323 (3)	
C2	0.2020 (3)	0.55970 (10)	0.20485 (13)	0.0397 (4)	
H2	0.3446	0.5575	0.2536	0.048*	
C3	0.1620 (3)	0.62241 (11)	0.12302 (14)	0.0462 (4)	
H3	0.2768	0.6629	0.1165	0.055*	
C4	−0.0502 (3)	0.62477 (11)	0.05064 (14)	0.0456 (4)	
H4	−0.0788	0.6668	−0.005	0.055*	
C5	−0.2179 (3)	0.56490 (11)	0.06128 (13)	0.0426 (4)	
H5	−0.3605	0.5664	0.0125	0.051*	
N6	−0.1782 (2)	0.50342 (8)	0.14226 (10)	0.0358 (3)	
O7	−0.33607 (19)	0.44536 (8)	0.15290 (11)	0.0507 (3)	
S8	0.04017 (6)	0.41513 (2)	0.31047 (3)	0.03918 (16)	
C9	0.3207 (3)	0.43634 (10)	0.39937 (14)	0.0415 (4)	
H9A	0.4475	0.4338	0.3522	0.05*	
H9B	0.319	0.4923	0.4327	0.05*	
C10	0.3594 (2)	0.37147 (9)	0.49432 (12)	0.0348 (3)	
C11	0.5483 (3)	0.31656 (10)	0.50101 (13)	0.0404 (4)	
H11	0.6517	0.3197	0.4462	0.049*	
C12	0.5852 (3)	0.25699 (10)	0.58820 (15)	0.0465 (4)	
H12	0.7138	0.2208	0.5919	0.056*	
C13	0.4330 (3)	0.25101 (11)	0.66927 (14)	0.0481 (4)	
H13	0.4565	0.2103	0.7271	0.058*	
C14	0.2452 (3)	0.30575 (13)	0.66418 (15)	0.0527 (4)	
H14	0.1426	0.3024	0.7194	0.063*	
C15	0.2084 (3)	0.36542 (11)	0.57771 (14)	0.0461 (4)	
H15	0.081	0.402	0.5752	0.055*	

### Atomic displacement parameters (Å^2^)

**Table:**

	*U*^11^	*U*^22^	*U*^33^	*U*^12^	*U*^13^	*U*^23^
C1	0.0293 (7)	0.0344 (7)	0.0321 (7)	0.0014 (5)	−0.0005 (5)	−0.0014 (5)
C2	0.0311 (7)	0.0454 (8)	0.0407 (8)	−0.0045 (6)	−0.0019 (6)	0.0043 (6)
C3	0.0422 (9)	0.0470 (9)	0.0482 (9)	−0.0091 (7)	0.0021 (7)	0.0095 (7)
C4	0.0481 (9)	0.0473 (9)	0.0397 (8)	0.0013 (7)	0.0004 (7)	0.0105 (7)
C5	0.0381 (8)	0.0479 (9)	0.0382 (8)	0.0026 (7)	−0.0073 (6)	0.0031 (7)
N6	0.0298 (6)	0.0378 (6)	0.0377 (6)	−0.0022 (5)	−0.0027 (5)	−0.0023 (5)
O7	0.0360 (6)	0.0504 (7)	0.0612 (7)	−0.0139 (5)	−0.0092 (5)	0.0073 (6)
S8	0.0355 (2)	0.0354 (2)	0.0437 (2)	−0.00537 (13)	−0.00524 (15)	0.00604 (14)
C9	0.0362 (8)	0.0388 (8)	0.0459 (8)	−0.0033 (6)	−0.0075 (6)	0.0058 (6)
C10	0.0342 (7)	0.0324 (7)	0.0355 (7)	−0.0012 (6)	−0.0035 (5)	−0.0015 (6)
C11	0.0370 (8)	0.0444 (8)	0.0397 (7)	0.0034 (6)	0.0044 (6)	−0.0004 (6)
C12	0.0460 (9)	0.0408 (8)	0.0502 (9)	0.0109 (7)	−0.0024 (7)	0.0025 (7)
C13	0.0590 (10)	0.0445 (9)	0.0374 (8)	−0.0046 (7)	−0.0054 (7)	0.0064 (7)
C14	0.0543 (10)	0.0660 (11)	0.0393 (8)	−0.0035 (9)	0.0121 (7)	0.0004 (8)
C15	0.0421 (9)	0.0505 (9)	0.0458 (8)	0.0107 (7)	0.0065 (7)	−0.0034 (7)

### Geometric parameters (Å, °)

**Table:**

C1—N6	1.3678 (18)	C9—H9A	0.97
C1—C2	1.381 (2)	C9—H9B	0.97
C1—S8	1.7450 (14)	C10—C11	1.383 (2)
C2—C3	1.376 (2)	C10—C15	1.389 (2)
C2—H2	0.93	C11—C12	1.383 (2)
C3—C4	1.382 (2)	C11—H11	0.93
C3—H3	0.93	C12—C13	1.373 (2)
C4—C5	1.369 (2)	C12—H12	0.93
C4—H4	0.93	C13—C14	1.378 (3)
C5—N6	1.355 (2)	C13—H13	0.93
C5—H5	0.93	C14—C15	1.378 (2)
N6—O7	1.3090 (16)	C14—H14	0.93
S8—C9	1.8205 (15)	C15—H15	0.93
C9—C10	1.505 (2)		
			
N6—C1—C2	119.53 (13)	C10—C9—H9B	109.9
N6—C1—S8	111.98 (10)	S8—C9—H9B	109.9
C2—C1—S8	128.49 (11)	H9A—C9—H9B	108.3
C3—C2—C1	120.09 (14)	C11—C10—C15	118.26 (14)
C3—C2—H2	120	C11—C10—C9	120.56 (14)
C1—C2—H2	120	C15—C10—C9	121.18 (14)
C2—C3—C4	119.47 (15)	C12—C11—C10	120.85 (14)
C2—C3—H3	120.3	C12—C11—H11	119.6
C4—C3—H3	120.3	C10—C11—H11	119.6
C5—C4—C3	119.71 (15)	C13—C12—C11	120.29 (15)
C5—C4—H4	120.1	C13—C12—H12	119.9
C3—C4—H4	120.1	C11—C12—H12	119.9
N6—C5—C4	120.65 (14)	C12—C13—C14	119.47 (15)
N6—C5—H5	119.7	C12—C13—H13	120.3
C4—C5—H5	119.7	C14—C13—H13	120.3
O7—N6—C5	121.37 (12)	C13—C14—C15	120.39 (16)
O7—N6—C1	118.08 (12)	C13—C14—H14	119.8
C5—N6—C1	120.55 (12)	C15—C14—H14	119.8
C1—S8—C9	99.76 (7)	C14—C15—C10	120.73 (15)
C10—C9—S8	108.78 (10)	C14—C15—H15	119.6
C10—C9—H9A	109.9	C10—C15—H15	119.6
S8—C9—H9A	109.9		
			
N6—C1—C2—C3	0.4 (2)	C2—C1—S8—C9	5.82 (16)
S8—C1—C2—C3	179.80 (13)	C1—S8—C9—C10	177.00 (11)
C1—C2—C3—C4	−0.4 (3)	S8—C9—C10—C11	117.36 (14)
C2—C3—C4—C5	0.1 (3)	S8—C9—C10—C15	−63.08 (17)
C3—C4—C5—N6	0.1 (3)	C15—C10—C11—C12	0.2 (2)
C4—C5—N6—O7	−179.46 (15)	C9—C10—C11—C12	179.81 (14)
C4—C5—N6—C1	−0.1 (2)	C10—C11—C12—C13	0.5 (3)
C2—C1—N6—O7	179.21 (13)	C11—C12—C13—C14	−1.0 (3)
S8—C1—N6—O7	−0.28 (16)	C12—C13—C14—C15	0.8 (3)
C2—C1—N6—C5	−0.2 (2)	C13—C14—C15—C10	0.0 (3)
S8—C1—N6—C5	−179.65 (11)	C11—C10—C15—C14	−0.5 (2)
N6—C1—S8—C9	−174.73 (11)	C9—C10—C15—C14	179.91 (15)

### Hydrogen-bond geometry (Å, °)

**Table:**

*D*—H···*A*	*D*—H	H···*A*	*D*···*A*	*D*—H···*A*
C5—H5···O7^i^	0.93	2.42	3.323 (2)	164
C14—H14···Cg1^ii^	0.93	2.92	3.560 (2)	127
C4—H4···Cg2^iii^	0.93	2.99	3.777 (2)	143

Symmetry codes: (i) −*x*−1, −*y*+1, −*z*; (ii) −*x*, −*y*+1, −*z*+1; (iii) −*x*, *y*+1/2, −*z*+1/2.
